# TP53 Mutations in AML Patients Are Associated with Dismal Clinical Outcome Irrespective of Frontline Induction Regimen and Allogeneic Hematopoietic Cell Transplantation

**DOI:** 10.3390/cancers15123210

**Published:** 2023-06-16

**Authors:** Davidson Zhao, Mojgan Zarif, Qianghua Zhou, José-Mario Capo-Chichi, Andre Schuh, Mark D. Minden, Eshetu G. Atenafu, Rajat Kumar, Hong Chang

**Affiliations:** 1 Department of Laboratory Medicine and Pathobiology, University of Toronto, Toronto, ON M5S 1A8, Canada; 2 Department of Laboratory Hematology, Laboratory Medicine Program, University Health Network, Toronto, ON M5G 2C4, Canada; 3 Department of Medical Oncology and Hematology, Princess Margaret Cancer Centre, University Health Network, Toronto, ON M5G 2M9, Canada; 4 Department of Biostatistics, University Health Network, Toronto, ON M5G 2C4, Canada; 5 Hans Messner Allogeneic Blood and Marrow Transplantation Program, Division of Medical Oncology and Hematology, Princess Margaret Cancer Centre, University Health Network, Toronto, ON M5G 2M9, Canada

**Keywords:** *TP53*, next-generation sequencing, AML, transplantation

## Abstract

**Simple Summary:**

*TP53* mutations are adverse-risk genetic aberrations in acute myeloid leukemia (AML). The optimal treatment approach in patients with *TP53*-mutated (*TP53*^MUT^) AML remains unclear. We aimed to evaluate the prognostic implications of different frontline treatment strategies and transplantation for patients with *TP53*^MUT^ AML. Patients treated with intensive induction or azacitidine-venetoclax induction had no significant improvement in survival compared to patients treated with other HMA regimens despite having higher complete remission rates. Transplantation was not significantly associated with improved outcomes in time-dependent or landmark analysis, however, transplanted patients with lower *TP53*^MUT^ variant allele frequency (VAF) at the time of diagnosis had superior outcomes compared to transplanted patients with higher *TP53* VAF. Current therapeutic strategies remain ineffective for *TP53*^MUT^ AML patients, which highlights the urgent need for new treatment strategies for this high-risk population.

**Abstract:**

*TP53* mutations are associated with extremely poor outcomes in acute myeloid leukemia (AML). The outcomes of patients with *TP53*-mutated (*TP53*^MUT^) AML after different frontline treatment modalities are not well established. Allogeneic hematopoietic cell transplantation (allo-HCT) is a potentially curative procedure for AML; however, long-term outcomes among patients with *TP53*^MUT^ AML after allo-HCT are dismal, and the benefit of allo-HCT remains controversial. We sought to evaluate the outcomes of patients with *TP53*^MUT^ AML after treatment with different frontline induction therapies and allo-HCT. A total of 113 patients with *TP53*^MUT^ AML were retrospectively evaluated. Patients with *TP53*^MUT^ AML who received intensive or azacitidine-venetoclax induction had higher complete remission rates compared to patients treated with other hypomethylating-agent-based induction regimens. However, OS and EFS were not significantly different among the induction regimen groups. Allo-HCT was associated with improved OS and EFS among patients with *TP53*^MUT^ AML; however, allo-HCT was not significantly associated with improved OS or EFS in time-dependent or landmark analysis. While the outcomes of all patients were generally poor irrespective of therapeutic strategy, transplanted patients with lower *TP53*^MUT^ variant allele frequency (VAF) at the time of diagnosis had superior outcomes compared to transplanted patients with higher *TP53* VAF. Our study provides further evidence that the current standards of care for AML confer limited therapeutic benefit to patients with *TP53* mutations.

## 1. Introduction

The *TP53* gene, located on chromosome 17p, plays a central role in anti-tumour response by regulating the cell cycle and apoptosis [1]. Mutations in *TP53* occur in approximately 10–20% of acute myeloid leukemia (AML) patients and are associated with an extremely poor prognosis [2]. *TP53* mutation is an adverse-risk genetic abnormality according to the 2022 ELN risk classification of AML due to its association with complex karyotype, advanced age, and resistance to standard therapies [3]. *TP53* mutation defines a distinct standalone entity within the International Consensus Classification of Myeloid Neoplasms and Acute Leukemia [4]. 

In fit patients with high-risk AML, the standard treatment approach is induction therapy, followed by allogeneic hematopoietic cell transplantation (allo-HCT) as a curative modality for those in remission [3]. Patients with *TP53*-mutated (*TP53*^MUT^) AML have a lower probability of achieving remission when treated with intensive chemotherapy and, consequently, have poor outcomes; for those who achieve remission, allo-HCT provides a relatively small improvement in long-term survival [2,5,6,7,8,9]. 

Given that there is limited data comparing the outcomes after different treatment modalities for patients with *TP53*^MUT^ AML, we retrospectively reviewed our institution’s experience with *TP53*^MUT^ AML. We assessed the prognostic implications of different frontline treatment strategies and transplantation for patients with *TP53*^MUT^ AML.

## 2. Materials and Methods

### 2.1. Patients and Treatment

We screened all adult University Health Network (UHN) patients who were diagnosed with AML with blasts ≥20% from February 2015 to December 2021 and who had molecular testing results available. Patients’ medical records were retrospectively assessed with respect to demographic and clinicopathological data, treatment modalities, and survival outcomes. The study was approved by the University Health Network Research Ethics Board. 

Fifty-seven patients with *TP53*^MUT^ AML received intensive induction therapy, with regimens including fludarabine, cytarabine, idarubicin, and granulocyte-colony stimulating factor (FLAG-IDA) (n = 32); cytarabine and daunorubicin (3 + 7) (n = 23); and liposomal cytarabine and daunorubicin (CPX-351) (n = 2). The dosages of these induction regimens are provided in the Supplementary Materials. Thirty-one patients received HMA-based induction therapy, with regimens including azacitidine and venetoclax (AZA-VEN) (n = 10), azacitidine alone (n = 18), guadecitabine (n = 2), and azacitidine and enasidenib (n = 1). The latter three treatment regimens were referred to as “other HMA-based regimens”. Patients treated with HMA-based regimens received a median of 3 (range, 1–12) cycles of HMA. Patients treated with FLAG-IDA induction who achieved complete remission were subsequently treated with FLAG-IDA consolidation. Patients treated with cytarabine and daunorubicin who achieved complete remission proceeded to be treated with cytarabine and daunorubicin consolidation or high-dose cytarabine (HiDAC) consolidation. 

According to the American Society of Transplantation and Cellular Therapy recommendations for unfavourable-risk AML, allo-HCT was offered to patients with *TP53*^MUT^ AML in CR if a suitable donor was identified [10,11]. Patients received 1–2 cycles of consolidation chemotherapy before proceeding to treatment with allo-HCT. 

### 2.2. Mutational Analysis and Determination of TP53 Allelic Status

Next-generation sequencing (NGS) was performed using a custom gene panel of 49 myeloid genes (Oxford Gene Technologies, Oxford, UK) or 54 myeloid genes (Illumina, San Diego, CA, USA) and run on the MiSeq platform (Illumina), as previously described [12,13,14]. The limit of detection for variant calling was 2%. For both panels, for 13/41 genes, the complete coding regions were analyzed, and for 28/41 genes, the same exonic hotspots were analyzed (Tables S1 and S2). All consensus-coding *TP53* exons were interrogated. Interpretation and classification of variants was performed as previously described [12]. Briefly, pathogenic (AMP/ASCO Tier 1) variants consisted of (a) loss-of-function truncating variants (nonsense, frameshift, and splicing) and (b) missense or in-frame variants (typically, within the DNA-binding domain) demonstrating loss-of-function (e.g., p.R110P) [15,16]. Likely pathogenic (AMP/ASCO Tier 2) variants consisted of (a) missense or in-frame variants involving the same residue (e.g., R110) as an established pathogenic variant and (b) missense or in-frame variants (typically, within the DNA binding domain) classified as disruptive in the TP53 IARC database [17]. Variants of uncertain significance (VUS, AMP/ASCO Tier 3) included variants classified as partially or fully transcriptionally active according to the TP53 IARC database as well as variants with no functional evidence of deleterious potential. VUS and benign polymorphisms were excluded from analysis. When patients had multiple mutations in the same gene, the higher variant allele frequency (VAF) was used for analysis. *TP53* mutations were visualized using cBioPortal [18,19].

Patients were considered to be double/multi-hit *TP53* when (A) at least two *TP53* variants were detected via NGS, (B) one *TP53* variant detected via NGS co-occurred with cytogenetic loss of *TP53,* or(C) one *TP53* variant was detected via NGS with a VAF of ≥55%, as previously described [20]. 

### 2.3. Karyotype Analysis

In accordance with the International System for Human Cytogenetic Nomenclature’s guidelines, karyotypes were obtained from diagnostic bone marrow samples and described as appropriate. The cytogenetic loss of *TP53* was determined as previously described [21]. 

### 2.4. Statistical Analysis

Categorical variables were summarized as counts and percentages. Continuous variables were summarized as medians and ranges. Evaluation of patient outcome including overall survival (OS) and event-free survival (EFS) was carried out through retrospective analysis of patient records. OS was calculated from the date of diagnosis to last follow-up or death. EFS was calculated from the date of diagnosis to last follow-up, relapse, or death. Log-rank test was used to compare Kaplan–Meier survival curves. Univariate Cox proportional-hazard regression models were fitted to identify prognostic factors for OS and EFS. Cox proportional-hazard regression was used to evaluate the prognostic impact of transplantation as a time-dependent variable, as transplant (if any) can occur during the follow-up period. Differences in categorical variables were assessed using the Fisher’s exact test. Differences in numerical variables across different groups were assessed using the Kruskal–Wallis and Wilcoxon rank-sum tests. Statistical analysis was performed using R version 4.0.5 (R Core Team (2020). R: A language and environment for statistical computing, Vienna, Austria). 

## 3. Results

### 3.1. Patient Characteristics

We identified 113 patients with *TP53*^MUT^ AML from our institution. Patient characteristics of the cohort after being stratified by frontline treatment modality are summarized and compared in Table 1. Fifty-seven (50%) patients received intensive chemotherapy, ten (9%) patients received AZA-VEN, twenty-one (19%) patients received other HMA-based regimens, and twenty-five (22%) patients received best supportive care. Gender ratio, baseline hematological parameters, and clinical ontogeny did not differ among the different treatment groups. However, patients who were treated with intensive chemotherapy were younger than those who were treated with AZA-VEN, other HMA-based regimens, and best supportive care (*p* = 0.0050, *p* < 0.0001, and *p* < 0.0001, respectively). In addition, the complete remission rate in the intensive chemotherapy and AZA-VEN groups were higher compared to the other HMA-based regimens group (*p* = 0.0013 and *p* = 0.022, respectively). A total of seventeen patients received allo-HCT after achieving remission; fifteen of the transplanted patients were treated with intensive induction chemotherapy, and two of the transplanted patients were treated with AZA-VEN induction. Eight patients had a matched unrelated donor transplant, five patients had a matched related donor transplant, and four patients had a haploidentical donor transplant. The median time-to-transplantation from diagnosis was 4.11 months (range 2.1–6.7 months). 

Additional baseline characteristics of the cohort are summarized in Table S3. According to the WHO 2016 classification, the majority of patients were classified as either AML with myelodysplasia-related changes (n = 92, 81%) or therapy-related AML (n = 13, 12%). The most frequent co-mutations were detected in *DNMT3A* (n = 17, 15%), *IDH1* (n = 11, 10%), *TET2* (n = 8, 7%), and *JAK2* (n = 5, 4%). The most frequent cytogenetic abnormalities were complex karyotype (n = 95, 84%), −5/del(5q) (n = 69, 61%), −7/del(7q) (n = 52, 46%), and -17/del(17p)/dic(17p) (n = 49, 43%). 

**Figure 1:**
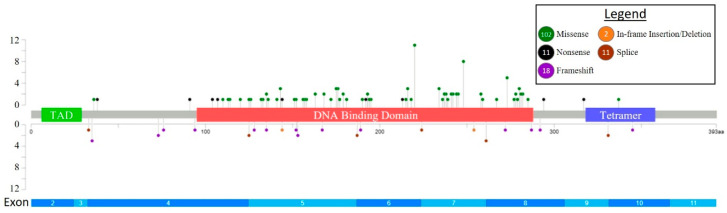
Distribution and frequency of *TP53* mutations in the cohort. Lolliplot indicates the distribution and frequency of *TP53* missense, nonsense, frameshift, in-frame deletions/insertions, and splice site mutations.

A total of 144 *TP53* mutations were detected, including 102 (71%) missense mutations, 18 (13%) frameshift mutations, 11 (8%) nonsense mutations, 11 (8%) splice site mutations, and 2 (1%) in-frame amino acid insertions/deletions (Figure 1). 

### 3.2. Outcomes of Patients According to Treatment and Molecular Characteristics

**Figure 2:**
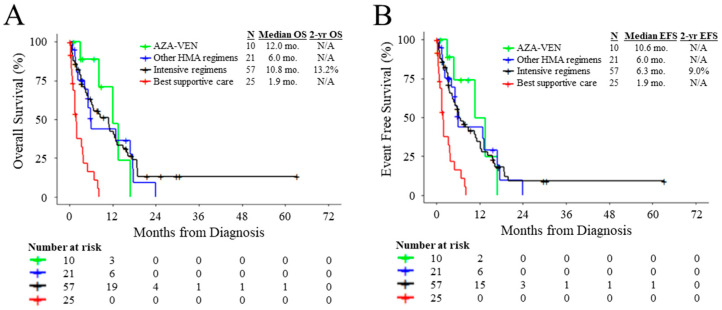
Outcomes according to frontline treatment modality. Kaplan–Meier plot for (**A**) OS and (**B**) EFS of patients with *TP53*^MUT^ AML stratified by frontline treatment regimen.

Compared to patients with *TP53*^MUT^ AML receiving best supportive care, patients who were treated with intensive induction, AZA-VEN, or other HMA-based regimens had superior OS (HR: 0.23, 95% CI: 0.12–0.42, *p* < 0.0001; HR: 0.07, 95% CI: 0.02–0.34, *p* = 0.0009; and HR: 0.25, 95% CI: 0.12–0.55, *p* = 0.0005, respectively) and EFS (HR: 0.28, 95% CI: 0.15–0.49, *p* < 0.0001; HR: 0.10, 95% CI: 0.02–0.42, *p* = 0.0020; and HR: 0.25, 95% CI: 0.12–0.55, *p* = 0.0005, respectively) (Figure 2A,B). Patients with *TP53*^MUT^ AML who received intensive regimens did not have significantly different OS or EFS compared to patients treated with AZA-VEN (HR: 1.20, 95% CI: 0.47–3.07, *p* = 0.70; and HR: 1.39, 95% CI: 0.55–3.53, *p* = 0.48, respectively) or other HMA-based regimens (HR: 0.84, 95% CI: 0.47–1.53, *p* = 0.58; and HR: 1.01, 95% CI: 0.56–1.82, *p* = 0.96, respectively) (Figure 2A,B). Patients with *TP53*^MUT^ AML treated with AZA-VEN did not have significantly different OS or EFS compared to patients treated with other HMA-based regimens (HR: 0.82, 95% CI: 0.29–2.35, *p* = 0.71; and HR: 0.79, 95% CI: 0.28–2.22, *p* = 0.65, respectively) (Figure 2A,B). Overall, while patients benefited from treatment compared to best supportive care, no treatment strategy conferred significantly superior outcomes in our cohort. 

Patients treated with HMA-based and intensive induction regimens were included in all downstream survival analyses. No significant difference in OS or EFS was observed when stratifying patients by the presence or absence of mutation in the DNA binding domain of *TP53* (HR: 0.94, 95% CI: 0.43–2.06, *p* = 0.869; and HR: 0.94, 95% CI: 0.43–2.06, *p* = 0.873, respectively) (Figure S1A,B). We further evaluated if any of the frequently co-mutated genes were associated with differences in OS or EFS. Among the treated patients, *DNMT3A* and *TET2* mutations were associated with marginally inferior OS (HR: 2.02, 95% CI: 0.99–4.13, *p* = 0.055; and HR: 2.40, 95% CI: 0.94–6.10, *p* = 0.066 respectively) and EFS (HR: 1.81, 95% CI: 0.89–3.68, *p* = 0.103; and HR: 2.32, 95% CI: 0.92–5.87, *p* = 0.076, respectively). The presence of an *IDH1* mutation was not associated with significantly different OS or EFS (HR: 0.73, 95% CI: 0.29–1.84, *p* = 0.508; and HR: 0.61, 95% CI: 0.24–1.54, *p* = 0.298, respectively). 

**Figure 3:**
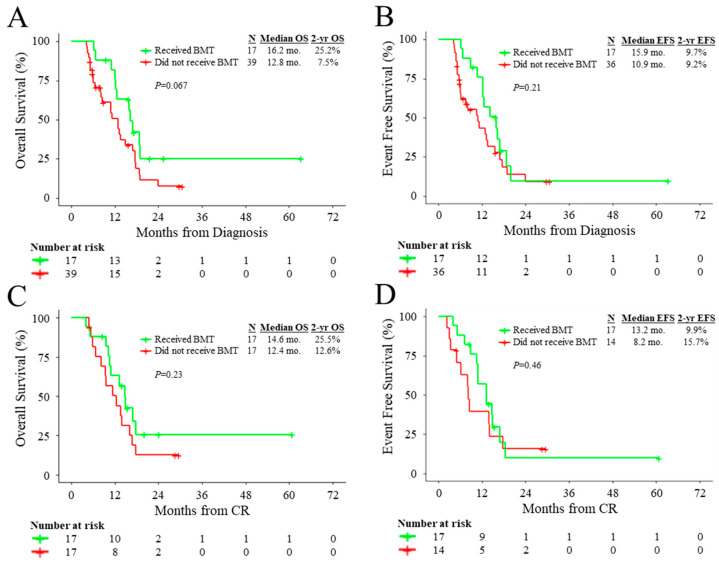
Outcomes according to allo-HCT status in landmark analysis. (**A**,**B**) Landmark analysis for OS and EFS from diagnosis stratified by allo-HCT status. (**C**,**D**) Landmark analysis for OS and EFS from achievement of CR stratified by allo-HCT status.

In patients treated with HMA-based and intensive induction regimens, allo-HCT was associated with improved OS (HR: 0.39, 95% CI: 0.20–0.75, *p* = 0.0047) and EFS (HR: 0.44, 95% CI: 0.24–0.82, *p =* 0.010) compared to non-transplanted patients (Figure S2A,B). However, when evaluating the impact of transplantation as a time-dependent variable, transplantation was not significantly associated with improved OS (HR: 0.61, 95% CI: 0.30–1.25, *p* = 0.18) or EFS (HR: 0.74, 95% CI: 0.38–1.47, *p* = 0.39). Landmark analysis for OS that excluded patients who died or were lost to follow-up before 4.11 months following diagnosis (corresponding to the median time from diagnosis to allo-HCT) indicated that allo-HCT marginally improved OS (HR: 0.53, 95% CI: 0.26–1.06, *p* = 0.072) (Figure 3A). Similarly, landmark analysis for EFS that excluded patients who died, were lost to follow-up, or relapsed before 4.11 months following diagnosis indicated that allo-HCT did not significantly improve EFS (HR: 0.65, 95% CI: 0.33–1.27, *p* = 0.21) (Figure 3B). 

To evaluate whether patients benefited from allo-HCT after the achievement of CR, we performed a separate landmark analysis of patients that achieved CR. Landmark analysis for OS, which excluded patients who died or were lost to follow-up before 2.34 months following achievement of CR (corresponding to the median time from achievement of CR to allo-HCT), indicated that allo-HCT did not significantly improve OS (HR: 0.62, 95% CI: 0.28–1.37, *p* = 0.24) in remission (Figure 3C). Similarly, landmark analysis for EFS that excluded patients who died, were lost to follow-up, or relapsed before 2.34 months following the achievement of CR indicated that allo-HCT did not significantly improve EFS (HR: 0.74, 95% CI: 0.33–1.67, *p* = 0.47) in remission (Figure 3D). 

**Figure 4:**
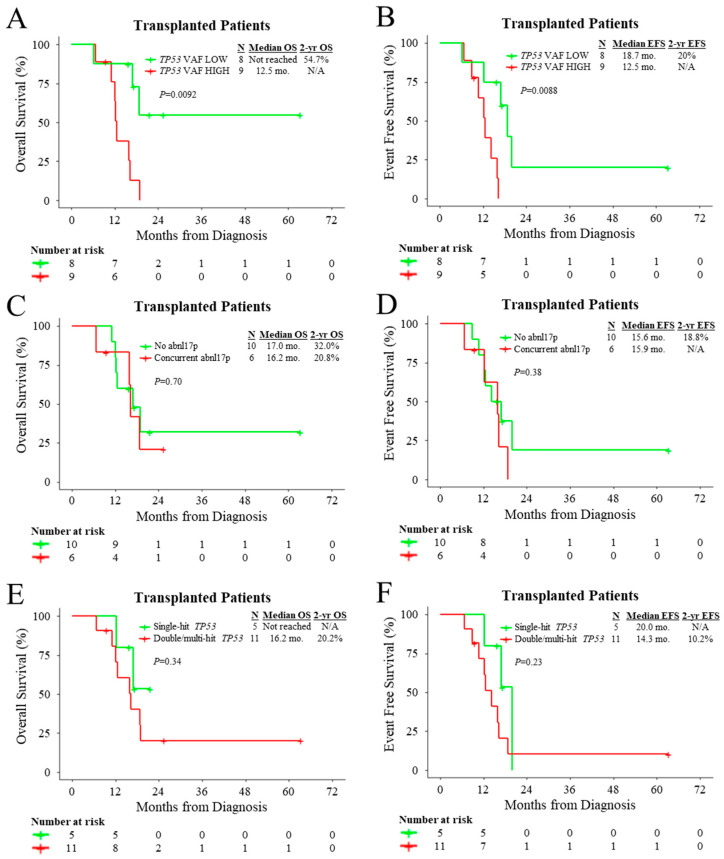
Outcomes of transplanted patients according to molecular and cytogenetic characteristics. Kaplan–Meier plot for OS and EFS of transplanted patients with *TP53*^MUT^ AML stratified by (**A**,**B**) median *TP53* variant allele frequency (45%), (**C**,**D**) presence or absence of concurrent cytogenetic loss of *TP53*, and (**E**,**F**) *TP53* allelic status.

Given the dismal outcomes of patients with *TP53*^MUT^ AML treated with allo-HCT, we sought to identify factors that may predict favourable outcomes with respect to transplantation. When using the median *TP53* VAF of transplanted patients (45%) as the cut-off, transplanted patients with low *TP53* VAF at the time of diagnosis had superior OS (HR: 0.19, 95% CI: 0.05–0.74, *p* = 0.017) and EFS (HR: 0.15, 95% CI: 0.03–0.75, *p* = 0.020) compared to those with high *TP53* VAF (Figure 4A,B). Among transplanted patients, concurrent cytogenetic abnormality in 17p did not modify OS (HR: 1.29, 95% CI: 0.36–4.61, *p* = 0.70) or EFS (HR: 1.70, 95% CI: 0.51–5.61, *p* = 0.39) (Figure 4C,D). In addition, double/multi-hit *TP53* status did not significantly influence OS (HR: 2.11, 95% CI: 0.44–10.03, *p* = 0.35) or EFS (HR: 2.20, 95% CI: 0.59–8.24, *p* = 0.24) among transplanted patients (Figure 4E,F). 

To further confirm that the favourable outcome associated with transplantation may be confined to patients with low *TP53* mutational burden, we performed subgroup landmark analyses for treated patients stratified by the median *TP53* VAF of the transplanted patients (45%). For patients with low *TP53* VAF, landmark analysis for OS that excluded patients who died or were lost to follow-up before 4.11 months following diagnosis (corresponding to the median time from diagnosis to allo-HCT) indicated that allo-HCT significantly improved OS (HR: 0.21, 95% CI: 0.06–0.73, *p* = 0.014) (Figure S3A). Similarly, landmark analysis for EFS that excluded patients who died, were lost to follow-up, or relapsed before 4.11 months following diagnosis indicated that allo-HCT marginally improved EFS (HR: 0.36, 95% CI: 0.13–1.01, *p* = 0.053) among patients with low *TP53* VAF (Figure S3B). In contrast, for patients with high *TP53* VAF, landmark analysis for OS that excluded patients who died or were lost to follow-up before 4.11 months following diagnosis indicated that allo-HCT did not significantly improve OS (HR: 1.10, 95% CI: 0.44–2.71, *p* = 0.84) (Figure S3C). Similarly, landmark analysis for EFS that excluded patients who died, were lost to follow-up, or relapsed before 4.11 months following diagnosis indicated that allo-HCT did not significantly improve EFS (HR: 1.06, 95% CI: 0.41–2.71, *p* = 0.91) among patients with high *TP53* VAF (Figure S3D).

## 4. Discussion

In this study, we report a single-institution evaluation of different treatment modalities for patients with *TP53*^MUT^ AML. While the use of any treatment was associated with better outcomes than best supportive care, intensive induction chemotherapy was not associated with a better outcome than HMA therapy. Furthermore, while transplanted patients had significantly superior outcomes compared to non-transplanted patients, this association was lost in time-dependent analysis and in landmark analysis. 

Anti-cancer responses to cytotoxic chemotherapies are critically dependent on wild-type *TP53*, as the activation of p53 by chemotherapy-induced DNA damage induces apoptosis and cancer cell death [22,23]. As such, standard induction treatments using DNA-damaging drugs may be a suboptimal therapeutic approach for patients with *TP53*^MUT^ AML. Prior clinical studies have demonstrated that patients with *TP53*^MUT^ AML have poor outcomes and response rates when treated with intensive chemotherapy regimens [24,25,26,27,28,29]. In addition, other less-intensive therapies such as hypomethylating agents and venetoclax have shown varying levels of efficacy among *TP53*^MUT^ AML patients [30,31,32,33]. In our cohort, patients with *TP53*^MUT^ AML had no significant differences in outcomes after treatment with intensive and non-intensive frontline regimens despite the younger age of the intensively treated patients. While the addition of venetoclax significantly improved complete remission rates among patients with *TP53*^MUT^ AML treated with HMA-based regimens, survival was not significantly improved with venetoclax, which is in line with prior reports [34,35,36]. Indeed, *TP53* mutations have been shown to confer resistance to BCL-2 inhibition in preclinical studies [37,38,39]. Clinical trials for novel targeted agents and immunotherapeutic approaches such as *TP53*^MUT^ re-activators and anti-CD47 blockade have shown potential in the frontline setting of *TP53*^MUT^ AML and should be explored further in this high-risk population [40]. 

Allo-HCT is considered to be a potentially curative option for high-risk AML patients; however, whether transplantation confers a beneficial impact among patients with *TP53*^MUT^ AML is controversial. Prior studies have largely supported the use of allo-HCT in these patients as they have reported superior outcomes in transplanted patients compared to non-transplanted patients [27,35,41,42].Conversely, there is evidence in the literature that patients with *TP53*^MUT^ AML do not significantly benefit from allo-HCT [34]. We previously evaluated the impact of allo-HCT among patients with AML with myelodysplasia-related changes and reported that allo-HCT had a significant benefit among *TP53*^MUT^ patients [43]. However, consistent with our data presented herein, *TP53*^MUT^ patients did not experience sustained and plateauing long-term outcomes and survival rates after allo-HCT. Since the outcomes of transplanted and non-transplanted patients may be confounded due to guarantee-time bias, we performed a prognostic evaluation of allo-HCT using time-dependent and landmark analyses [44]. In these analyses, *TP53*^MUT^ patients who received allo-HCT did not have significantly superior outcomes compared to non-transplant patients, suggesting that the differences in outcome according to allo-HCT status are confounded by guarantee-time bias. Despite this, among transplanted patients, low *TP53* VAF at the time of diagnosis was associated with superior outcomes. Further landmark analysis of patient subgroups revealed that the favourable outcome associated with transplant is confined to patients with low *TP53* VAF, who had significantly improved outcomes with allo-HCT compared to non-transplanted patients with low *TP53* VAF. Our findings are consistent with previous reports demonstrating associations between low *TP53* VAF and superior outcomes in transplanted patients with *TP53*^MUT^ AML [35,45]. As such, *TP53* VAF may have clinical utility for guiding the decision-making process of whether *TP53*^MUT^ patients should undergo transplantation. While other studies have shown an adverse prognostic impact of cytogenetic loss of *TP53* among transplanted patients with *TP53*^MUT^ AML, we did not observe a significant difference in our cohort [46]. Future studies are needed to optimize transplant approaches and post-transplant monitoring practices and to explore the prognostic benefit of allo-HCT for *TP53*^MUT^ AML patients. 

The updated 2022 ELN guidelines for the management of AML recommend for *TP53* mutational analysis to be performed within the first cycle of chemotherapy [3]. At our institution, *TP53* sequencing is performed via NGS, which has a relatively longer turnaround time compared to other diagnostic tests. The lack of benefit for intensive chemotherapy among patients with *TP53*^MUT^ AML suggests that genetic results for *TP53* should be obtained before beginning treatment to avoid ineffective and aggressive therapy. Rapid and cost-effective methods such as immunohistochemistry have shown potential for predicting *TP53* mutational status and should be further explored in the clinical setting [47,48]. 

We acknowledge that our study has several limitations. First, our study is retrospective in nature and includes limited patient numbers in its subgroup analyses. Future multi-center prospective studies are needed to confirm our data. Second, transplanted patient outcomes may be influenced by factors such as patient performance status, conditioning regimen intensity, and post-transplant graft-versus-host disease. Further studies are needed to evaluate the impact of these factors on patient outcomes and to determine the optimal allo-HCT approach for patients with *TP53*^MUT^ AML. Third, this study only focused on patients with *TP53*^MUT^ AML; future prospective studies should include patients with and without *TP53* mutations to evaluate the clinical impact of *TP53* mutations across different treatment groups.

## 5. Conclusions

In conclusion, outcomes among patients with *TP53*^MUT^ AML after intensive therapy and allo-HCT remain poor; however, patients with low *TP53* VAF at the time of diagnosis may still benefit from transplantation. Our study provides further evidence that patients with *TP53*^MUT^ AML derive limited benefit from current treatments and highlights the urgent need for new treatment strategies.

## Figures and Tables

**Table 1:** Baseline characteristics of the *TP53*^MUT^ AML cohort.

Clinical Feature	Total (n = 113)	Intensive Induction (n = 57)	AZA-VEN (n = 10)	Other HMA Regimens (n = 21)	Supportive Care (n = 25)	*p*-Value
Age (y), median [range]	70.7 [34.3–91.7]	63.7 [34.3–76.7]	72.0 [55.2–83.1]	78.8 [60.2–91.7]	77.6 [53.8–91.6]	<0.0001 ^a^
Male/Female gender	52:61	32:25	6:4	6:15	8:17	0.052 ^b^
WBC count ×10^9^/L, median [range]	2.9 [0.3–76.9]	2.9 [0.3–76.9]	2.6 [0.3–11.2]	2.6 [1.1–16.8]	4.0 [0.8–72.4]	0.322 ^a^
Platelets ×10^9^/L, median [range]	46 [5–782]	46 [7–782]	48 [10–162]	68 [5–290]	40 [6–338]	0.981 ^a^
Hemoglobin, ×10^12^/L, median [range]	82 [57–125]	85 [60–125]	86 [68–118]	82 [67–121]	79 [57–106]	0.358 ^a^
BM blasts %, median [range]	37 [20–90]	44 [20–90]	50 [23–90]	32 [20–79]	32 [20–83]	0.285 ^a^
LDH, IU/L, median [range]	320 [128–3391]	336 [128–2790]	309 [157–770]	285 [148–841]	371 [199–3391]	0.081 ^a^
Clinical ontogeny						
de novo AML	83 (73)	46 (81)	7 (70)	15 (71)	15 (60)	0.054 ^b^
s-AML	13 (12)	7 (12)	1 (10)	0 (0)	5 (20)	
t-AML	17 (15)	4 (7)	2 (20)	6 (29)	5 (20)	
Complete remission	36 (41)	29 (51)	5 (50)	2 (10)	n/a	0.0022 ^b^

Abbreviations: WBC, white blood cell; BM, bone marrow; LDH, lactate dehydrogenase; s-AML, secondary AML evolving from antecedent hematological disorder; t-AML, therapy-related AML. ^a^ Kruskal–Wallis test; ^b^ Fisher’s exact test.

## Data Availability

Data are available on request from the corresponding author.

## Supplementary Materials

The following supporting information can be downloaded at: https://www.mdpi.com/article/10.3390/cancers15123210/s1, Figure S1: Outcomes according to presence/absence of *TP53* mutations in the DNA binding domain. Figure S2: Outcomes according to transplant status. Figure S3: Outcomes according to TP53 VAF and transplant status; Table S1: Gene panel for targeted sequencing; Table S2: Exon coverage for hotspot genes; Table S3: Summary of additional clinicopathological features.
