# Supplementary material for: TP53 Mutations in AML Patients Are Associated with Dismal Clinical Outcome Irrespective of Frontline Induction Regimen and Allogeneic Hematopoietic Cell Transplantation
Source: Cancers (Basel). 2023 Jun 16;15(12):3210. doi: 10.3390/cancers15123210 (PMC10296444; doi:10.3390/cancers15123210)
Supplement: Supplementary file 1 [file cancers-15-03210-s001.zip › cancers-2404828-supplementary.pdf]

**Supplementary Materials**

**Intensive induction dosage**

FLAG-IDA: G-CSF 300 µg daily day 0-5, Idarubicin 10 mg/m$^2$ daily day 1-3, Fludarabine 30 mg/m$^2$ daily day 1-5, Cytarabine 2 g/m$^2$ daily day 1-5

7+3: Daunorubicin 60 mg/m$^2$ daily day 1-3, Cytarabine 200 mg/m$^2$ daily day 1-7, Cytarabine dose reduction to 100 mg/m$^2$ for patients age > 60 years

CPX-351 (Vyxeos): 44 mg/m$^2$ day 1, 3 and 5

**Supplementary Figure S1.** (A) OS and (B) EFS of treated *TP53*$^{MUT}$ patients stratified by presence or absence of *TP53* mutation within its DNA binding domain (amino acids 95-288).

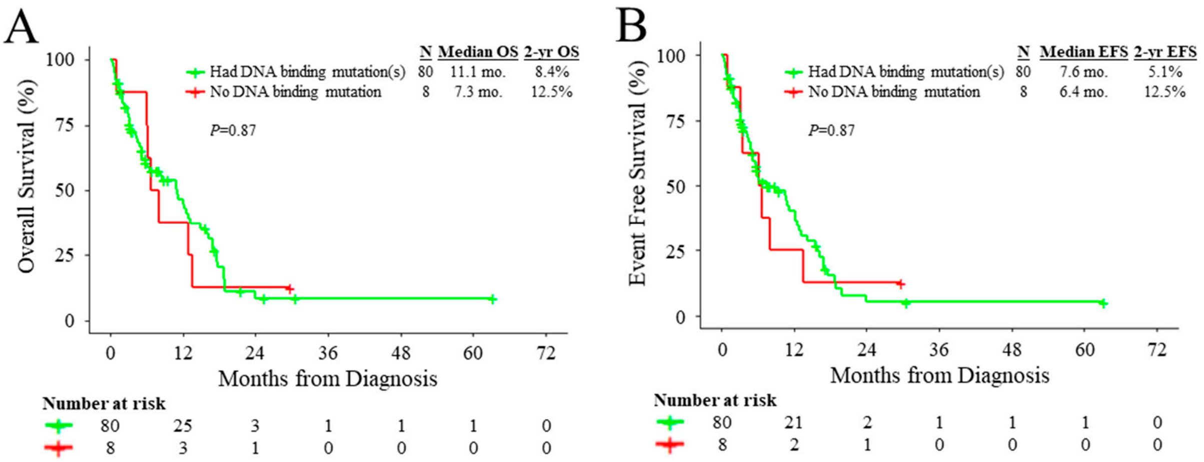

**Supplementary Figure S2.** (A) OS and (B) EFS of all treated *TP53*$^{MUT}$ patients stratified by allo-HCT status.

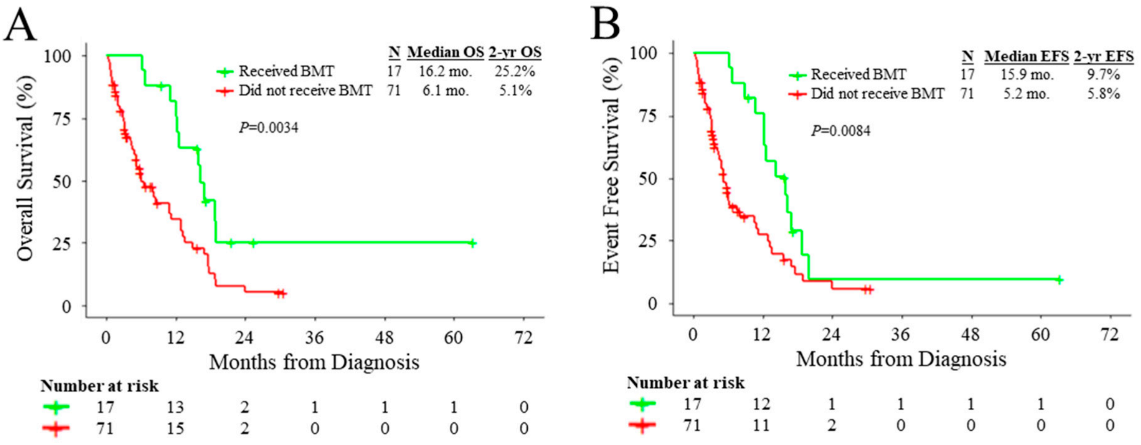

**Supplementary Figure S3.** (A-B) Landmark analysis for OS and EFS for patients with low *TP53* VAF (< 45%) stratified by allo-HCT status. (C-D) Landmark analysis for OS and EFS for patients with high *TP53* VAF (≥ 45%) stratified by allo-HCT status.

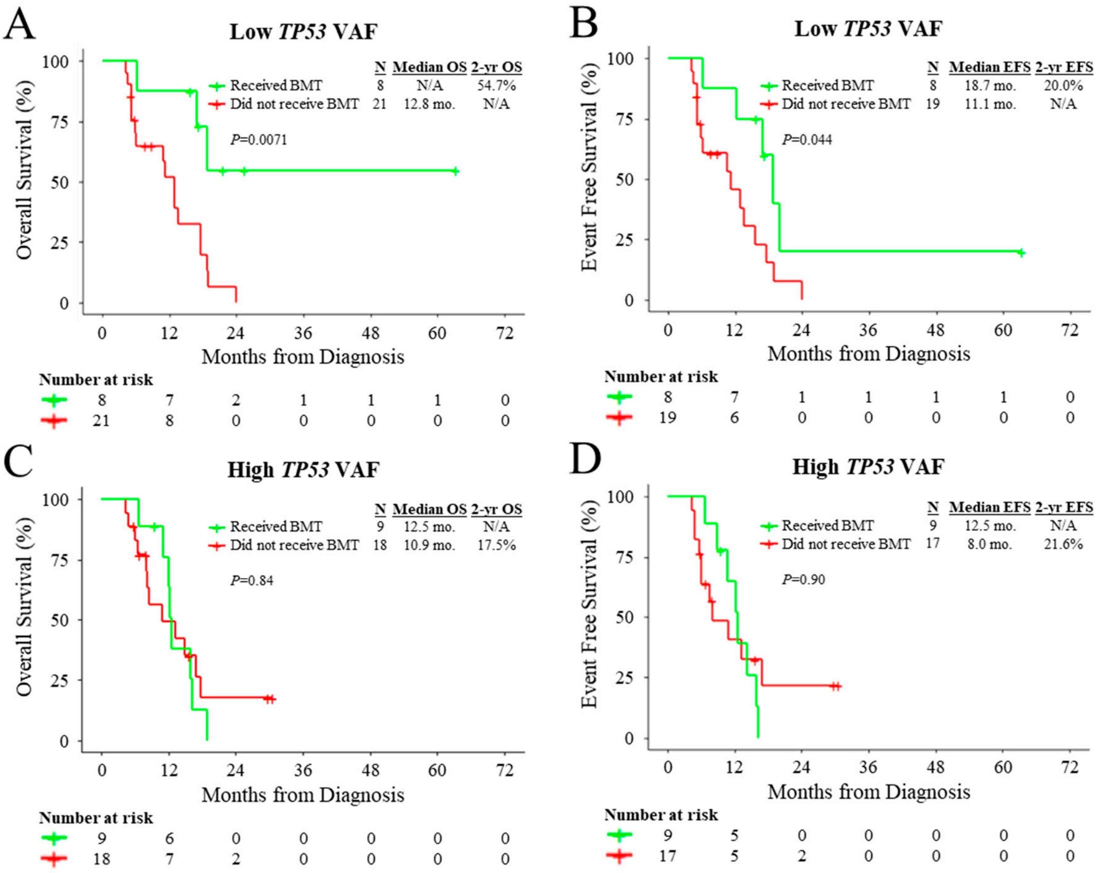

**Supplementary Table S1.** Gene panel for targeted sequencing

| Complete coding region coverage (13/41) | Hotspot coverage (28/41) |
|---|---|
| *BCOR* | *ASXL1* |
| *BCORL1* | *BRAF* |
| *CEBPA* | *CALR* |
| *CUX1* | *CBL* |
| *DNMT3A* | *CSF3R* |
| *ETV6* | *FBXW7* |
| *EZH2* | *FLT3* |
| *IKZF1* | *GATA2* |
| *PHF6* | *GNAS* |
| *RAD21* | *IDH1* |
| *RUNX1* | *IDH2* |
| *STAG2* | *JAK2* |
| *ZRSR2* | *KIT* |
| | *KMT2A* |
| | *KRAS* |
| | *MPL* |
| | *MYD88* |
| | *NOTCH1* |
| | *NPM1* |
| | *NRAS* |
| | *PTPN11* |
| | *SETBP1* |
| | *SF3B1* |
| | *SRSF2* |
| | *TET2* |
| | *TP53* |
| | *U2AF1* |
| | *WT1* |

| **Supplementary Table S2.** Exon coverage for hotspot genes | |
|---|---|
| **Gene** | **Exon Coverage** |
| *ASXL1* | 12 |
| *BRAF* | 15 |
| *CALR* | 9 |
| *CBL* | 8, 9 |
| *CSF3R* | 14-17 |
| *FBXW7* | 9-11 |
| *FLT3* | 14, 15, 20 |
| *GATA2* | 2-6 |
| *GNAS* | 8, 9 |
| *IDH1* | 4 |
| *IDH2* | 4 |
| *JAK2* | 12, 14 |
| *KIT* | 2, 8-11, 13, 17 |
| *KMT2A* | 5-8 |
| *KRAS* | 2,3 |
| *MPL* | 10 |
| *MYD88* | 3-5 |
| *NOTCH1* | 26-28, 34 |
| *NPM1* | 12 |
| *NRAS* | 2, 3 |
| *PTPN11* | 3, 13 |
| *SETBP1* | 4 |
| *SF3B1* | 13-16 |
| *SRSF2* | 1 |
| *TET2* | 3-11 |
| *TP53* | 2-11 |
| *U2AF1* | 2, 6 |
| *WT1* | 7, 9 |

| **Supplemental Table S3.** Summary of additional clinicopathological features | |
|---|---|
| Clinical feature | |
| AML subtype (WHO-HAEM4), *n* | |
| AML with myelodysplasia-related changes | 92 |
| Therapy-related AML | 13 |
| AML, NOS | 3 |
| AML with mutated *NPM1* | 2 |
| AML with inv(3)(q21.3q26.2) or t(3;3)(q21.3;q26.2); *GATA2*, *MECOM* | 1 |
| AML with inv(16)(p13.1q22) or t(16;16)(p13.1;q22); *CBFB-MYH11* | 1 |
| AML with mutated *RUNX1* (provisional entity) | 1 |
| Co-mutations, *n* (VAF range) | |
| *DNMT3A* | 17 (5-44) |
| *IDH1* | 11 (6-39) |
| *TET2* | 8 (5-46) |
| *JAK2* | 5 (7-48) |
| *CEBPA* | 4 (10-97) |
| *RUNX1* | 4 (22-54) |
| *ASXL1* | 4 (19-31) |
| *IDH2* | 4 (36-44) |
| *BCOR* | 3 (6-44) |
| *CUX1* | 3 (7-34) |
| *NPM1* | 3 (4-30) |
| *NRAS* | 3 (10-40) |
| *SF3B1* | 3 (31-42) |
| *SRSF2* | 3 (39-46) |
| *U2AF1* | 3 (19-43) |
| *STAG2* | 2 (18-29) |
| *CALR* | 2 (15-32) |
| *FLT3* | 2 (5-13) |
| *KIT* | 2 (24-49) |
| *KRAS* | 2 (12-19) |
| *MPL* | 2 (17-36) |
| *PTPN11* | 2 (34-34) |
| *BCORL1* | 1 (39) |
| *ETV6* | 1 (17) |
| *EZH2* | 1 (52) |
| *CSF3R* | 1 (5) |
| *FBXW7* | 1 (8) |
| *GATA2* | 1 (6) |
| *GNAS* | 1 (8) |
| *NOTCH1* | 1 (51) |
| *WT1* | 1 (54) |
| Common cytogenetic abnormalities, *n* | |
| Complex karyotype | 95 |
| -5/del(5q) | 69 |
| -7/del(7q) | 52 |
| -17/del(17p)/dic(17p) | 49 |
